# Case Report of Congenital Hepatoblastoma With the Onset at 30-Weeks' Gestation

**DOI:** 10.3389/fped.2022.905089

**Published:** 2022-07-01

**Authors:** Zheng Yan, Wei Bai, Li Li, Shuo Li, Ying Hua, Xiao-xiao Zhang, Xin-lin Hou

**Affiliations:** ^1^Department of Pediatrics, Peking University First Hospital, Beijing, China; ^2^Department of Obstetrics and Gynecology, Peking University First Hospital, Beijing, China

**Keywords:** alpha-fetoprotein (AFP), chemotherapy, congenital hepatoblastoma, recurrence, surgery

## Abstract

This study reports a case of hepatoblastoma with onset at 30-weeks' gestation and rapid growth rate. The postnatal enhanced CT confirmed an intrahepatic mass with a size of 8.5 cm × 6.6 cm and a clear boundary accompanied by uneven enhancement, displacement, and narrow lumen of the hepatic vein due to compression. The alpha-fetoprotein (AFP) at birth was 1,002,632 ng/ml (normal level 48,406 [±34,718] ng/ml). A diagnosis of congenital hepatoblastoma was established based on the imaging and laboratory outcomes. The infant received chemotherapy of Cisplatin-5 fluorouracil-Vincristine (C5V) on the fourth day after birth. After four courses of C5V, a complete tumor resection was performed, and the postoperative pathology was consistent with mixed epithelial and mesenchymal hepatoblastoma. Four more courses of C5V and one course of C5VD (C5V plus doxorubicin) followed the surgery. Infectious diarrhea and acute kidney injury (stage I) occurred during chemotherapy, which recovered after anti-infection and symptomatic treatment. The patient is currently 2 years old and still in complete remission. In this case, the onset of hepatoblastoma was early, and the tumor grew rapidly, resulting in an obvious compression effect. Chemotherapy was started early after birth, and the curative effect was satisfactory, suggesting that the hepatoblastoma based on clinical diagnosis with rapid tumor progression and severe dysfunction of surrounding organs caused by compression should undergo chemotherapy as soon as possible if a pathological diagnosis cannot be obtained temporarily, which also plays an important role in improving the complete resection rate of intraoperative tumor and reducing the recurrence rate of postoperative tumor.

## Introduction

Hepatoblastoma is the most common primary malignant liver tumor among children and is an embryo-derived tumor. Among children up to 5 years old, the incidence of hepatoblastoma is 1.2–1.5 per million children per year (1). Most occur in infancy with an incidence of 11.2 per million infants per year and about 8 to 10 times higher than in the first five years of life (2). Congenital hepatoblastoma is diagnosed during the fetal period and 3 months after birth; it is usually found by ultrasound with a description that there is a heterogeneous hyperechoic mass in the liver with a clear margin, and blood flow signals can be detected. Postnatal clinical diagnosis can be made by combining abdominal mass, alpha-fetoprotein, liver damage, and typical imaging features, while histological diagnosis needs depend on pathological examination. This disease can occur insidiously during the fetal period with rapid progress, while the fetus appears normal except for mild anemia sometimes at early birth. In later stages, jaundice, ascites, fever, anemia, poor appetite, weight loss, and visible abdominal varicose veins will appear; the large mass inside the abdominal cavity may also lead to respiratory distress. Approximately 20% of patients have distant metastasis at diagnosis; accordingly, early detection and treatment are crucial. The Pediatric Oncology Group in the United States and the International Pediatric Liver Oncology Strategy Group in Europe recommend surgery combined with chemotherapy as the standard mode of treatment for hepatoblastoma (3, 4). Preoperative and postoperative chemotherapy significantly improves children's prognosis, producing a 5-year disease-free and event-free survival rate of around 70% (1). However, due to the particularity of growth and development, pharmacokinetics, and pharmacodynamics at this stage, the initiation and tolerance of chemotherapy for congenital hepatoblastoma children diagnosed in the intrauterine and neonatal period have not been agreed upon (5). This case study reports on a congenital hepatoblastoma with an intrauterine onset at 30-weeks' gestation. Preoperative chemotherapy was initiated 4 days after birth, followed by complete surgical resection of the tumor and continued chemotherapy after the surgery. This treatment was successful and provides a reference experience in the treatment of congenital hepatoblastoma.

## Case Description

**Figure 1:**
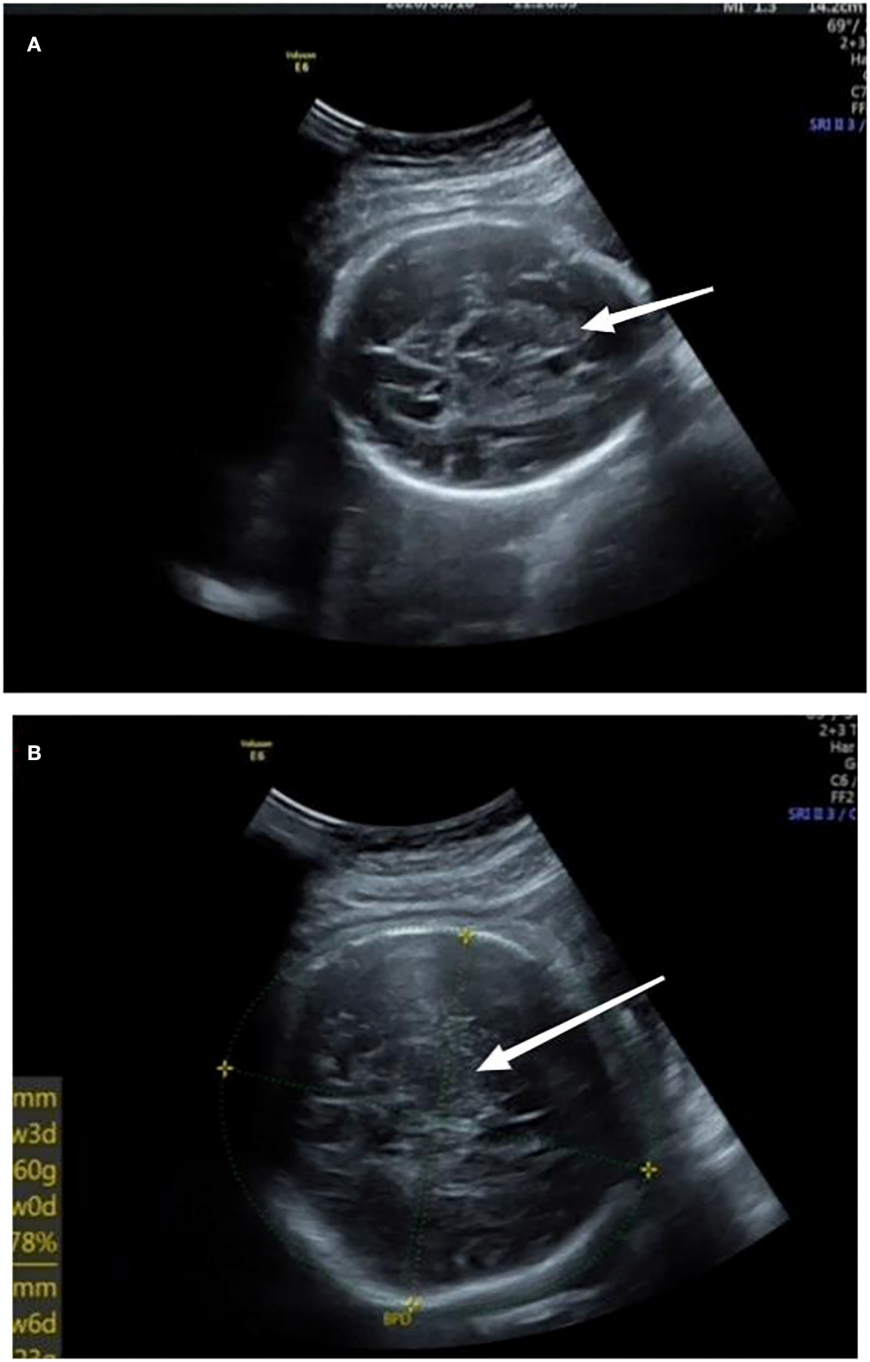
**(A)** At 30-weeks' gestation, there was a medium to high echoic mass in the parenchyma of the right lobe of liver with a size of 1.8 cm × 1.8 cm × 1.5 cm and a clear boundary surrounded by a low echoic dark halo; inside of the mass, a few strips of blood flow signals could be detected. **(B)** At 38-weeks' gestation, a mass located in the right lobe of the liver, which had increased to a size of 7.1 cm × 7.9 cm × 5.6 cm. Arrows referred to uneven echoes.

A 32-year-old primiparous pregnant woman's routine prenatal ultrasound at 30-weeks' gestation revealed a 1.8 cm × 1.8 cm × 1.5 cm medium-to-high echoic mass with a clear boundary surrounded by a low echoic dark halo in the fetus' parenchyma of the right lobe of the liver. Inside the mass, a few strips of blood flow signals could be detected (Figure 1A). A fetal MRI at 31-weeks' gestation showed a 1.8 cm × 1.5 cm × 1.2 cm nodule with a clear boundary and slightly high signal intensity on T2WI, slightly low signal intensity on TIWI, and slightly high signal intensity on DWI, suggesting a mass located in the right lobe of the liver. At 38-weeks' gestation, the mass had increased rapidly to a size of 7.1 cm × 7.9 cm × 5.6 cm (Figure 1B).

**Figure 2:**
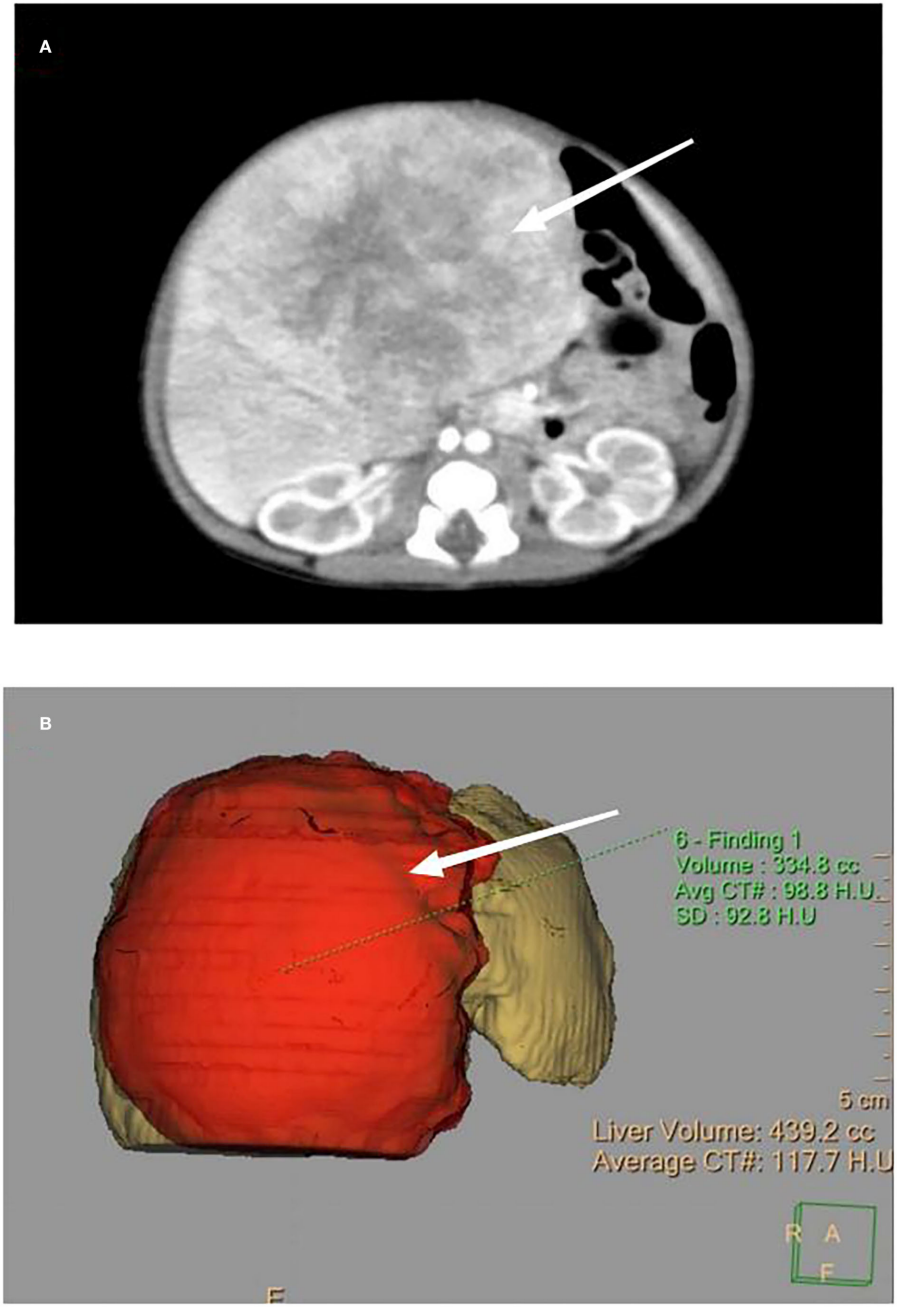
**(A)** Enhanced CT, as indicated by the arrow, showing a large, round mass with a size of 8.5 cm × 6.6 cm, a clear boundary, and uneven enhancement. **(B)** Three-dimensional model of the enhanced CT showing the appearance of the mass.

A female infant was delivered by cesarean section at 39 + 3-weeks' gestation with a birth weight of 3,040 g and Apgar scores of 10 for the 1st and 5th min. The infant suffered from poor appetite, frequent vomiting, yellow skin, and abdominal distension. On physical examination, the liver could be palpated 10 cm below the ribs; firm consistency, blunt edges, and a smooth surface were observed. Laboratory tests showed alanine aminotransferase (ALT) 380 IU/L, aspartate aminotransferase (AST) 315I U/L, total bilirubin (TBIL) 251 umol/L, direct bilirubin (DBIL) 165 umol/L, and fibrinogen (FIB) 1.2g/L; alpha-fetoprotein (AFP) was significantly increased to 1,002,632 ng/ml (normal 48,406 [± 34,718] ng/ml). An abdominal ultrasound on day 1 showed a mass of about 8.1 cm × 7.2 cm with mixed echoes, a clear margin, and detectable calcification inside. It was mainly located in the right anterior lobe. An abdominal enhanced CT and a three-dimensional model on day 2 showed a large round mass (8.5 cm × 6.6 cm), a clear boundary, and uneven enhancement (Figures 2A,B). The hepatic vein was displaced, and the lumen had become thinner because of compression from this mass. A bone puncture (marrow and biopsy) on day 3 after birth was normal, and no evidence of distant metastasis was found. Finally, a clinical diagnosis of congenital hepatoblastoma was reached.

Due to the large size and rapid growth of the tumor, the compression on the surrounding organs led to liver and coagulation dysfunction and feeding difficulties. Additionally, the tumor surface tension was so high that the risk of tumor rupture during biopsy was increased significantly. After full communication with the surgeon, it was clear that there were too many temporary contraindications for puncture and operation. After the parents understood the condition and situation of the infant, they all hoped to start chemotherapy as soon as possible to reduce the load of the tumor. Preoperative chemotherapy with Cisplatin-5fluorouracil-Vincristine (C5V) was initiated on day four. After four courses of chemotherapy, the tumor was reduced to 5.8 cm × 5.3 cm, and the AFP had declined to 2,516 ng/ml (normal 323 [± 278] ng/ml). During chemotherapy, infectious diarrhea and transient acute kidney injury (type I) occurred on the third day of the first chemotherapy remission. The highest serum creatinine was 1.55 mg/dl, urinary microalbumin 193 mg/L (normal 0–19 mg/L), urinary transferrin 9.48 mg/L (normal 0–2 mg/L), and urine α1 microglobulin 94.7 mg/L (normal 0–12 mg/L), indicating early kidney injury. These readings returned to normal after anti-infection and symptomatic treatment. Complete resection of the tumor in the right lobe and part of the left lobe of the liver was performed 3 months after birth. The tumor size was about 5.5 cm × 5 cm × 4 cm, and no intravascular tumor thrombus was found.

Postoperative pathology showed mixed epithelial and mesenchymal hepatoblastoma. The epithelial component was the fetal type which differentiated well. After surgery, four courses of C5V were continued, followed by one course of C5VD (C5V plus doxorubicin). There was no organ injury during chemotherapy, and no cardiotoxicity, hepatotoxicity, or neurotoxicity was detected. In terms of blood, platelet levels remained normal, and occasionally, granulocytopenia or anemia could be recovered. Abdominal enhanced CT re-examination showed that the tumor had disappeared completely. Postoperative chemotherapy was continued until 6 months after birth. After that, the patient had repeated abdominal ultrasounds and abdominal enhanced CT re-examinations at 6 months and 1 year old, and no evidence of recurrence was found. The patient's AFP gradually decreased to 18–32 ng/ml (normal 8.5 [±5.5] ng/ml). She is now 2 years old and is still receiving follow-up examinations (Table 1).

**Table 1:** Timeline of the episode of care.

**Time**	**Event**
30-weeks' gestation	The prenatal ultrasound found a 1.8 cm × 1.8 cm × 1.5 cm mass
38-weeks' gestation	The size of the mass had increased rapidly
30-weeks' gestation	The size of the mass increased to 7.1 cm × 7.9 cm × 5.6 cm
39 + ^3^-weeks' gestation	A female infant was delivered by cesarean section
Two days after birth	The size of the mass was 8.5 cm × 6.6 cm on CT
Four days after birth	Four cycles of C5V chemotherapy were started
Three months after birth	Complete resection of tumor was performed, and four cycles of C5V and a cycle of C5VD were administered after the surgery
2 years old	No evidence of recurrence was found

## Discussion

Congenital liver occupation is a rare occurrence, with hemangioma being the most common, followed by hepatoblastoma and mesenchymal hamartoma (6). Hepatoblastoma is an embryo-derived liver tumor with early onset and is concentrated in children under 5 years old. Although it is the most common hepatic malignancy, the proportion of congenital hepatoblastoma diagnosed in the neonatal period is less than 10% (7). Established risk factors include preterm birth, low birth weight, familial adenomatous polyposis, Beckwith-Wiedemann syndrome, and Trisomy 18 syndrome (3, 8–10). The gestational age of congenital hepatoblastoma cases found in utero ranges from 20 to 42 weeks, but prenatal diagnosis of hepatoblastoma accounts for <5% (11).

It has been reported that only 1 out of 42 congenital hepatoblastoma cases was diagnosed as intrauterine (7). Delayed diagnosis often delays postnatal treatment, leading to increased risk of liver mass compression, tumor rupture, distant metastasis, and a generally poor prognosis. Recent advances in fetal ultrasound and fetal MRI imaging have led to liver tumor imaging characteristics being established, resulting in steady increase in intrauterine diagnoses. A retrospective study on 194 cases of congenital liver tumors included 56 cases of intrauterine diagnosis, with a diagnostic rate of 29%. Moreover, the study included 32 liver tumor cases, of which 9 were intrauterine diagnosis, with a diagnosis rate of 28% (12). The current case study's fetal ultrasound at 30-weeks' gestation showed a heterogeneous hyperechoic mass in the right lobe of the liver with a clear boundary and abundant blood flow signals. The fetal MRI showed a nodule in the right lobe of the liver with high signal on T2WI, low signal on TIWI, and a clear boundary. If the MRI finds a nodule with a clear boundary, low signal on T1WI, high signal on T2WI, annular low signal shadow around, and occasional spot calcification, it is highly suggestive of hepatoblastoma (13).

Prognostic factors for hepatoblastoma include the tumor's PRETEXT stage, Evans stage, AFP level at diagnosis, and pathological classification. They are also associated with early detection and timely chemotherapy and surgical treatment before distant metastasis, occurrence of respiratory and heart failure and tumor rupture due to compression effects (14–16). To improve the prognosis of infants with hepatoblastoma, the importance of early treatment should be emphasized. In our case, we established a clinical diagnosis of hepatoblastoma by analyzing the abdominal mass, alpha-fetoprotein, liver damage, and typical imaging features, and ruling out hemangioma, mesenchymal hamartoma, neuroblastoma, and distant metastasis comprehensively. Considering the intrauterine onset and diagnosis, preoperative chemotherapy could be started early. However, the large size and high surface tension of the tumor made the risk of tumor rupture during biopsy remarkably increased. On the one hand, we could not obtain a tissue diagnosis in time; on the other hand, the tumor's rapid growth caused compression on the surrounding organs, which led to liver dysfunction and feeding difficulties. Consequently, after reaching an agreement with the parents, we started chemotherapy on the infant based on the clinical diagnosis on day 4 after birth. Four courses of preoperative chemotherapy shrank the tumor by nearly 30% and decreased the AFP significantly, which facilitated complete surgical resection.

The neonatal period is a period of fast growth. The development of fetuses' organ structures and functions is so different that the pharmacokinetics and pharmacodynamics for chemotherapy drugs are unstable. Additionally, the infants' tolerance to chemotherapy drugs and the forward impact on development make chemotherapy in newborns difficult (6). Four infants with hepatoblastoma who received chemotherapy in the neonatal period have been reported to have died; of these, two started chemotherapy on the basis of pulmonary hypertension and died of respiratory failure on their 23rd and 61st day, respectively (15, 17), while two died of multiple tumor metastases during chemotherapy in their 6th and 20th month, respectively (18, 19). In recent years, it has been reported that for newborns with congenital hepatoblastoma, under the condition of general stability, chemotherapy should be started as early as possible in the neonatal period, as the tolerance for chemotherapy is satisfactory (20–22). Trobaugh-Lotrario et al. reported that the prognosis of infants with congenital hepatoblastoma is not worse than that of older children (2).

In recent years, the combination of surgery and chemotherapy is recommended internationally for treatment of hepatoblastoma; this treatment can significantly improve the negative rate of tumor resection margin and improve the prognosis. Congenital hepatoblastoma has the characteristics of early onset, rapid progression, easy metastasis, and risk of tumor rupture and compression effect. Preoperative chemotherapy should be implemented early, once the diagnosis is made. Sometimes, if biopsy is not available for clinically diagnosed hepatoblastoma, which progresses rapidly, squeezes other organs severely, and has a high risk of rupture, we can also start chemotherapy to reduce the harm caused by the tumor and create opportunities for biopsy and surgery. Although the structure and function of neonatal organs grow and develop rapidly, resulting in the instability of pharmacokinetics of chemotherapy drugs, reports in recent years state that chemotherapy can be tolerated in the short and long terms when the general condition of infants is stable. The infant in our case report had a stable general condition, although infectious diarrhea and acute kidney injury (stage I) occurred during chemotherapy but returned to normal after anti-infection and symptomatic treatment. Occasionally, we detected neutropenia or deficiency anemia, but all of which were recoverable. We followed up this infant for 2 years, and no tumor recurrence or functional impairment of systems and organs was observed.

## Data Availability Statement

The original contributions presented in the study are included in the article/supplementary material, further inquiries can be directed to the corresponding author/s.

## Author Contributions

ZY wrote the manuscript. WB and LL collected the data and followed up the case. SL and YH supervised the management and follow-up of the case. All authors revised and approved the final version of the manuscript and agreed to be accountable for the content of the article.

## Funding

This research was supported by Beijing Municipal Science & Technology Commission (Z191100006619049).

## Conflict of Interest

The authors declare that the research was conducted in the absence of any commercial or financial relationships that could be construed as a potential conflict of interest.

## Publisher's Note

All claims expressed in this article are solely those of the authors and do not necessarily represent those of their affiliated organizations, or those of the publisher, the editors and the reviewers. Any product that may be evaluated in this article, or claim that may be made by its manufacturer, is not guaranteed or endorsed by the publisher.

## References

[1] Hafberg E Borinstein SC Alexopoulos SP. Contemporary management of hepatoblastoma. Curr Opin Organ Transplant. (2019) 24:113–7. 10.1097/MOT.0000000000000618 30762666

[2] Trobaugh-Lotrario AD Chaiyachati BH Meyers RL Häberle B Tomlinson GE Katzenstein HM. Outcomes for patients with congenital hepatoblastoma. Pediatr Blood Cancer. (2013) 60:1817–25. 10.1002/pbc.24655 23798361

[3] Trobaugh-Lotrario AD López-Terrada D Li P Feusner JH. Hepatoblastoma in patients with molecularly proven familial adenomatous polyposis: Clinical characteristics and rationale for surveillance screening. Pediatr Blood Cancer. (2018) 65:e27103. 10.1002/pbc.27103 29719120

[4] Katzenstein HM Furman WL Malogolowkin MH Krailo MD McCarville MB Towbin AJ. Upfront window vincristine/irinotecan treatment of high-risk hepatoblastoma: A report from the Children's Oncology Group AHEP0731 study committee. Cancer. (2017) 123:2360–7. 10.1002/cncr.30591 28211941 PMC5665173

[5] Ranganathan S Lopez-Terrada D Alaggio R. Hepatoblastoma and pediatric hepatocellular carcinoma: an update. Pediatr Dev Pathol. (2020) 23:79–95. 10.1177/1093526619875228 31554479

[6] Miura Y Saito J Shimanuki Y Takeyama J Murotsuki J. Diagnosis and treatment of a preterm infant with inoperable congenital hepatoblastoma–a case report. J Pediatr Hematol Oncol. (2015) 37:e188–90. 10.1097/MPH.0000000000000200 24942027

[7] Ergin H Yildirim B Dagdeviren E Yagci B Ozen F Sen N. Prenatally detected case of congenital hepatoblastoma. Pathol Oncol Res. (2008) 14:97–100. 10.1007/s12253-008-9001-8 18365769

[8] Oue T Kubota A Okuyama H Kawahara H Nara K Kawa K Kitajima H. Hepatoblastoma in children of extremely low birth weight: a report from a single perinatal center. J Pediatr Surg. (2003) 38:134–7. 10.1053/jpsu.2003.50027 12592636

[9] Valentin LI Perez L Masand P. Hepatoblastoma Associated with Trisomy 18. J Pediatr Genet. (2015) 4:204–6. 10.1055/s-0035-1565265 27617134 PMC4906528

[10] Mussa A Molinatto C Baldassarre G Riberi E Russo S Larizza L. Cancer risk in Beckwith-Wiedemann Syndrome: a systematic review and meta-analysis outlining a novel (Epi)genotype specific histotype targeted screening protocol. J Pediatr. (2016) 176:142–9.e1. 10.1016/j.jpeds.2016.05.038 27372391

[11] Sharma D Subbarao G Saxena R. Hepatoblastoma. Semin Diagn Pathol. (2017) 34:192–200. 10.1053/j.semdp.2016.12.015 28126357

[12] Isaacs H Jr. Fetal and neonatal hepatic tumors. J Pediatr Surg. (2007) 42:1797–803. 10.1016/j.jpedsurg.2007.07.047 18022426

[13] Birkemeier KL. Imaging of solid congenital abdominal masses: a review of the literature and practical approach to image interpretation. Pediatr Radiol. (2020) 50:1907–20. 10.1007/s00247-020-04678-1 33252758

[14] Varlas V Neagu O Moga A. Fetal pancreatic hamartoma associated with hepatoblastoma-an unusual tumor association. Diagnostics. (2022) 12:758. 10.3390/diagnostics12030758 35328311 PMC8947736

[15] Özdemir ZC Saglik AÇ Kar YD Köşger P Arik D Tekin AN. A case of congenital hepatoblastoma coexisting with pulmonary hypertension. Arch Iran Med. (2020) 23:621–3. 10.34172/aim.2020.73 32979909

[16] Yoo GH Mugarab-Samedi V Hansen G Miller G Givelichian L Kalaniti K. Rare cause of emergency in the first week of life: congenital hepatoblastoma (case report). Oxf Med Case Reports. (2020) 2020:omaa002. 10.1093/omcr/omaa002 32123567 PMC7037083

[17] Inoue A Suzuki R Urabe K Kawamura Y Masuda M Kishi K. Therapeutic experience with hepatoblastoma associated with trisomy 18. Pediatr Blood Cancer. (2018) 65:e27093. 10.1002/pbc.27093 29701292

[18] Kitanovski L Ovcak Z Jazbec J. Multifocal hepatoblastoma in a 6-month-old girl with trisomy 18: a case report. J Med Case Rep. (2009) 3:8319. 10.4076/1752-1947-3-8319 19830224 PMC2726543

[19] Kawasaki Y Makimoto M Samejima A Yoneda N Higashimoto K Soejima H. Hepatoblastoma in an extremely low birth-weight infant with Beckwith-Wiedemann syndrome. Pediatr Neonatol. (2018) 59:523–4. 10.1016/j.pedneo.2017.11.012 29203194

[20] Ofoegbu BN Abdel Salam SEE Diehl WG Ghosn L. Congenital hepatoblastoma in a growing health economy. BMJ Case Rep. (2019) 12:e223344. 10.1136/bcr-2017-223344 30898949 PMC6453373

[21] Zivot A Edelman M Glick R. Congenital Hepatoblastoma and Beckwith-Wiedemann Syndrome. J Pediatr Hematol Oncol. (2020) 42:e798–800. 10.1097/MPH.0000000000001565 31335825

[22] Huang LC Ho M Chang WC. Prenatal diagnosis of fetal hepatoblastoma with a good neonatal outcome: case report and narrative literature review. Pediatr Hematol Oncol. (2011) 28:150–4. 10.3109/08880018.2010.536299 21299342

